# Expression of nerve growth factor and heme oxygenase-1 predict poor survival of breast carcinoma patients

**DOI:** 10.1186/1471-2407-13-516

**Published:** 2013-11-01

**Authors:** Sang Jae Noh, Jun Sang Bae, Urangoo Jamiyandorj, Ho Sung Park, Keun Sang Kwon, Sung Hoo Jung, Hyun Jo Youn, Ho Lee, Byung-Hyun Park, Myoung Ja Chung, Woo Sung Moon, Myoung Jae Kang, Kyu Yun Jang

**Affiliations:** 1 Departments of Pathology, Research Institute of Clinical Medicine and Research Institute for Endocrine Sciences, Chonbuk National University Medical School, Jeonju, Republic of Korea; 2 Departments of Preventive Medicine, Chonbuk National University Medical School, Jeonju, Republic of Korea; 3 Departments of Surgery, Chonbuk National University Medical School, Jeonju, Republic of Korea; 4 Departments of Forensic Medicine, Chonbuk National University Medical School, Jeonju, Republic of Korea; 5 Departments of Biochemistry, Chonbuk National University Medical School, Research Institute for Endocrine Sciences, Jeonju, Republic of Korea

**Keywords:** Nerve growth factor, Heme oxygenase-1, Carcinoma, Breast

## Abstract

**Background:**

Nerve growth factor (NGF) is a neurotrophin and has been suggested to induce heme oxygenase-1 (HO1) expression. Although the role of HO1 in tumorigenesis remains controversial, recent evidence suggests NGF and HO1 as tumor-progressing factors. However, the correlative role of NGF and HO1 and their prognostic impact in breast carcinoma is unknown.

**Methods:**

We investigated the expression and prognostic significance of the expression of NGF and HO1 in 145 cases of breast carcinoma.

**Results:**

Immunohistochemical expression of NGF and HO1 was observed in 31% and 49% of breast carcinoma, respectively. The expression of NGF and HO1 significantly associated with each other, and both have a significant association with histologic grade, HER2 expression, and latent distant metastasis. The expression of NGF and HO1 predicted shorter overall survival of breast carcinoma by univariate and multivariate analysis. NGF expression was an independent prognostic indicator for relapse-free survival by multivariate analysis. The combined expression pattern of NGF and HO1 was also an independent prognostic indicator of overall survival and relapse-free survival. The patients with tumors expressing NGF had the shortest survival and the patients with tumor, which did not express NGF or HO1 showed the longest survival time.

**Conclusions:**

This study has demonstrated that individual expression of NGF or HO1, and the combined NGF/HO1 expression pattern could be prognostic indicators for breast carcinoma patients.

## Background

Nerve growth factor (NGF) is a neurotrophin, which shows neurotrophic activity on central and peripheral neuronal cells, and exerts variable effects on non-neuronal cells [1]. In addition to its neurotrophic effect, NGF is also known as a stimulator of cancer cell proliferation and tumor angiogenesis, and participates in tumor cell growth and invasion [1-3]. NGF is involved in the development and progression of many tumors of neural origin and epithelial tumors such as medulloblastoma, glioma, neuroblastoma, melanoma, pancreas cancer, prostate cancer, and breast carcinoma (BRCA) [1-4]. The main function of NGF is mediated by two membranes binding receptors: high affinity tyrosine kinase receptor TrkA and low affinity p75^NTR^[1-3]. In BRCA, NGF is shown to act as a mitogen for cancer cells through phosphorylation of TrkA, and it promotes survival and proliferation of cancer cells [2]. The expression of the NGF receptor (NGFR) TrkA enhanced the tumorigenic potential of BRCA in an animal model [5]. In addition, because blocking of NGF pathway was shown to have tumor-suppressive effects in BRCA, NGF was suggested as a potential therapeutic target for the treatment of BRCA [6-8].

Heme oxygenase-1 (HO1) is an enzyme that catalyzes heme breakdown, generating free iron, carbon monoxide, and bilirubin [9]. Because of the combined effect of its products, HO1 acts as a strong antioxidant with anti-inflammatory, anti-apoptotic, and immunomodulatory effects [10,11]. Therefore, HO1 is protective against various injuries, such as necrotizing enterocolitis [11] and ischemic-reperfusion injury [12]. However, anti-apoptotic and cytoprotective roles for chemotherapeutic agents targeting HO1 were shown to induce tumor-progression [13-15]. Increased expression of HO1 in malignant tissue compared with normal tissue has been reported in various human malignant tumors, such as prostate cancer [16], oral squamous cell carcinoma [17], and lung cancers [18,19]. However, there are conflicting reports regarding the prognostic role of HO1 in human malignant tumors. High expression of HO1 is associated with poor prognosis of non-small cell lung cancer [18]. In contrast, HO1 expression is associated with favorable prognosis of colorectal cancer patients [20] and low risk of lymph node metastasis in oral squamous cell carcinoma [21]. Therefore, the role of HO1 in human malignant tumors still remains controversial.

Increasing evidence suggests that NGF and HO1 are involved in tumorigenesis and could therefore be possible therapeutic targets of human malignant tumors. However, there are no previous reports examining the clinical significance of the expression of NGF itself in BRCA patients. In addition, both NGF and HO1 exert neuroprotective effects, and NGF induces HO1 expression via mitogen-activated protein kinase kinase activation [22] or in a phosphatidylinositol 3-kinase-dependent manner [23]. Therefore, there is a possibility that NGF and HO1 are cooperatively involved in the tumorigenesis via their roles in cellular adaptation to stress and resistance to apoptosis. However, the relationship between NGF and HO1 and the role of HO1 in cancer progression is still unclear in BRCA. Therefore, this study investigated the correlation between the expression of NGF and HO1 and their prognostic impact in BRCA.

## Methods

### Patients and tissue samples

One hundred and forty-five paraffin-embedded tissue samples from female BRCA patients who underwent wide local excision or modified radical mastectomy in Chonbuk National University Hospital from January 1997 to August 2002 were included in the present study. This study was approved by the institutional review board of Chonbuk National University Hospital. Informed consent was provided according to the Declaration of Helsinki.

The mean age at diagnosis of the 145 patients was 46.04 years (range: 22–72 years). Eighty-eight patients received modified radical mastectomy, and fifty-seven patients received breast conserving surgery. One hundred and twenty-nine patients received systemic adjuvant chemotherapy [CMF (cyclophosphamide, methotrexate and fluorouracil 5FU) chemotherapy or anthracycline- and taxane-based chemotherapy] and 121 patients received adjuvant endocrine therapy. One hundred and ten patients received both chemotherapy and endocrine therapy, and 5 patients received no adjuvant therapy. The median follow-up duration was 144.9 months (range, 7.7 - 192.6). Among the 145 BRCA patients, 21 patients experienced local relapse, 33 patients had latent distant metastasis, and 44 patients died from BRCA at the follow-up endpoint. The median survival was 192.0 months and the five- and ten-year survival rates for the entire BRCA patients were 81% and 74%, respectively. All the cases were reviewed and classified by two pathologists (KY Jang and SJ Noh) according to the World Health Organization Classification [24], and pathologic staging as reviewed in the 7th edition of the American Joint Committee on Cancer staging system [25]. The histologic diagnoses of 145 cases of BRCA were 137 invasive ductal carcinomas and 8 invasive lobular carcinomas. The patients were grouped according to the age, TNM stage, histologic type, modified Bloom and Richardson histologic grade (tubule and gland formation, nuclear pleomorphism and mitotic counts) [24], presence of local relapse, distant metastasis, and immunohistochemical expression of human epidermal growth factor receptor 2 (HER2), ER and PR.

### Immunohistochemical staining and scoring

Immunohistochemical staining was performed using 3.0 mm tumor cores for tissue microarray (TMA). To establish the TMA, we reviewed all of the H&E slides and took two 3.0 mm tissue cores from the paraffin-embedded tissue blocks per case at the area of highest tumor grade. An antigen retrieval procedure was performed with sodium citrate buffer using a microwave oven for 20 minutes. The following markers were used: NGF (1:200, Abcam, Cambridge, UK) and HO1 (1:200, Enzo Life Sciences, PA, USA). Immunohistochemical staining for NGF and HO1 were evaluated by the sum of the staining intensity scores and the staining area scores in each TMA core [26,27]. The staining intensity was scored as 0 (no staining), 1 (weak staining), 2 (intermediate staining), and 3 (strong staining). The staining area was scored as 0 (no staining cells), 1 (1% of the cells stained positive), 2 (2-10% of the cells stained positive), 3 (11-33% of the cells stained positive), 4 (34-66% of the cells stained positive), and 5 (66-100% of the cells stained positive). Thereafter, the combined score (obtained by adding the sum of the scores of two different TMA cores) was used for further analysis. The maximum combined score was 16 and the minimum sum score was zero. Subsequently, the expression of NGF and HO1 were grouped as positive or negative by receiver operating characteristic curve analysis at the highest positive likelihood ratio point for the death of BRCA patients. The cut-off point for NGF expression was 9 and was 14 for HO1 expression. The expression of NGF was considered positive when a combined score was greater or equal to nine and HO1 expression was considered positive when a combined score was greater than or equal to fourteen. HER2 immunostaining was considered positive if 30% or more of the tumor cell showed strong complete membrane staining. Immunostaining for estrogen receptor (ER) and progesterone receptor (PR) were considered positive if 1% or more of the tumor cells showed nuclear staining. Immunohistochemical scoring was performed by two pathologists (KY Jang and SJ Noh) who were blinded to the clinicopathologic information of the patients.

### Statistical analysis

The relationships between NGF and HO1 expression and other clinicopathological factors were determined using the Pearson’s chi-square test. The primary point of interest was overall survival (OS) and relapse-free survival (RFS). The follow-up endpoint was the date of death or the date of last contact through December 2012. OS duration was measured as the time from diagnosis to date of death from BRCA and the patients who were alive at last contact or died from other causes were treated as censored. RFS was calculated as the time from diagnosis to the date of relapse, death from BRCA, or last contact. Patients who were alive at last contact or died from other causes and who did not experience the relapse were treated as censored for RFS analysis. Univariate and multivariate Cox regression hazard analysis were performed to estimate the impact of clinicopathologic factors and expression of each marker on OS and RFS. Kaplan-Meier survival analysis with a log-rank test was used to illustrate the cumulative survival curve for OS and RFS. Statistical analyses were calculated using SPSS statistical software (IBM, version 18.0, CA, USA). *P*-values less than 0.05 were considered to be statistically significant.

## Results

### NGF and HO1 expression and its correlations with clinicopathologic factors of BRCA patients

**Figure 1:**
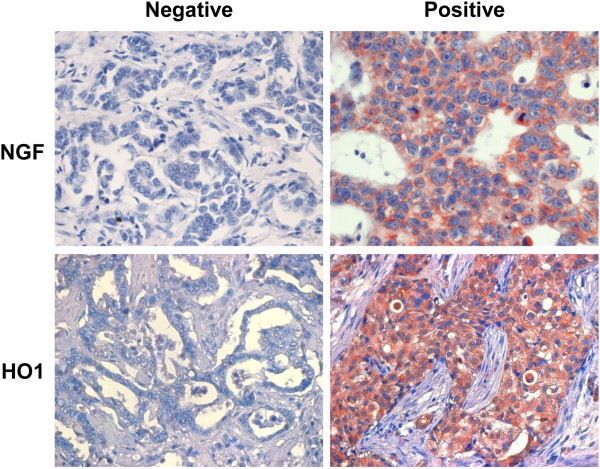
**Immunohistochemical expression of NGF and HO1 in breast carcinoma.** Original magnification, x400.

The expression of NGF and HO1 was seen mainly in the cytoplasm of tumor cells, and the expression of NGF and HO1 was grouped positive in 31% (45/145 of cases) and 49% (71/145) of BRCA samples, respectively (Figure 1). The expression of NGF was significantly associated with age (*P* = 0.035), histologic grade (*P* = 0.020), presence of latent distant metastasis (*P* = 0.004), and the expression of HER2 (*P* = 0.002) and ER (*P* = 0.005). Especially, a strong positive correlation between NGF and HO1 was found (*P* < 0.001). The expression of HO1 was significantly correlated with age (*P* = 0.029), histologic grade (*P* = 0.017), presence of latent distant metastasis (*P* < 0.001), and HER2 expression (*P* < 0.001) (Table 1).

**Table 1:** Association of the expression of NGF and HO1 with clinicopathological factors

**Characteristics**		**No.**	**NGF**		**HO1**	
			**Positive**	***P***	**Positive**	***P***
Age, y	<50	104	27 (26%)	0.035	45 (43%)	0.029
	≥50	41	18 (44%)		26 (63%)	
TNM stage	I	29	9 (31%)	0.836	17 (59%)	0.507
	II	97	28 (29%)		45 (46%)	
	III and IV	19	7 (37%)		9 (47%)	
T stage	1	41	10 (24%)	0.424	22 (54%)	0.675
	2	95	31 (33%)		44 (46%)	
	3 and 4	9	4 (44%)		5 (56%)	
LN metastasis	Absence	83	26 (31%)	0.930	39 (47%)	0.582
	Presence	62	19 (31%)		32 (52%)	
Latent distant metastasis	Absence	112	28 (25%)	0.004	45 (40%)	< 0.001
	Presence	33	17 (52%)		26 (79%)	
Histologic type	Ductal	137	43 (31%)	0.704	67 (49%)	0.952
	Lobular	8	2 (25%)		4 (50%)	
Histologic grade	1	49	9 (18%)	0.020	20 (41%)	0.017
	2	67	22 (33%)		30 (45%)	
	3	29	14 (48%)		21 (72%)	
HER2	Negative	105	25 (24%)	0.002	42 (40%)	< 0.001
	Positive	40	20 (50%)		29 (73%)	
ER	Negative	65	28 (43%)	0.005	35 (54%)	0.289
	Positive	80	17 (21%)		36 (45%)	
PR	Negative	60	23 (38%)	0.110	34 (57%)	0.119
	Positive	85	22 (26%)		37 (44%)	
HO1	Negative	74	5 (7%)	< 0.001		
	Positive	71	40 (56%)			

### Expression of NGF and HO1 correlates with overall survival and relapse-free survival in BRCA according to univariate analysis

**Figure 2:**
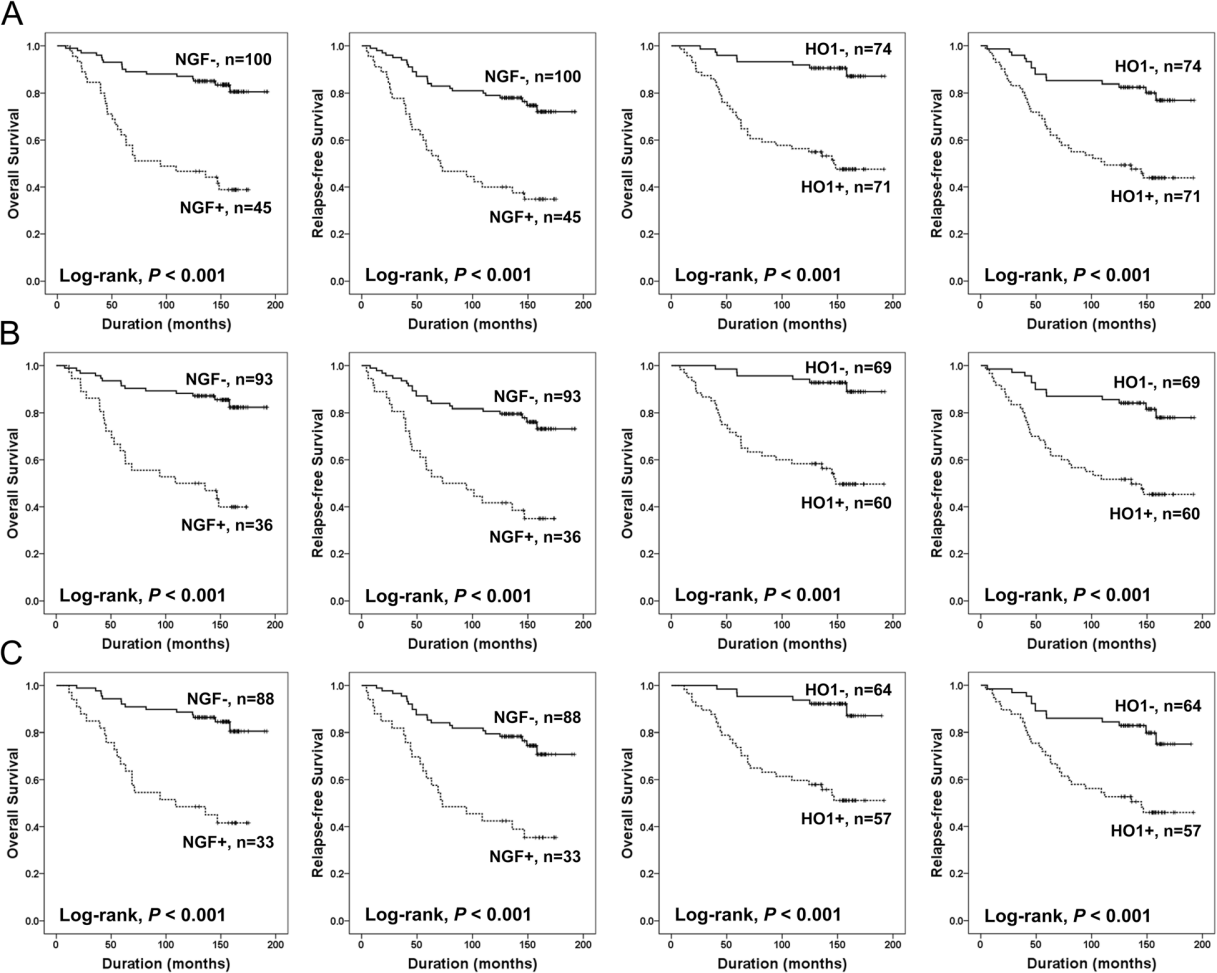
**Kaplan-Meier survival analysis according to the expression of NGF and HO1. A.** Overall survival (OS) and relapse-free survival (RFS) in 145 breast carcinoma (BRCA) patients. **B.** OS and RFS in 129 BRCA patients who received adjuvant chemotherapy. **C.** OS and RFS in 121 BRCA patients who received post-operative endocrine therapy.

Univariate survival analyses of the expression of NGF and HO1 and clinicopathological factors for OS and RFS are listed in Table 2. In the 145 BRCA patients, age of the patients (Log-rank, OS; *P* < 0.001, RFS; *P* = 0.017), HER2 expression (Log-rank, OS; *P* < 0.001, RFS; *P* < 0.001), NGF expression (Log-rank, OS; *P* < 0.001, RFS; *P* < 0.001), and HO1 expression (Log-rank, OS; *P* < 0.001, RFS; *P* < 0.001) were significantly associated with shorter OS and RFS (Figure 2A). The patients with NGF expression had a 4.674-fold (95% CI, 2.541-8.598) greater risk of death (*P <* 0.001) and its expression significantly associated with shorter RFS (*P* < 0.001, HR; 3.550, 95% CI; 2.074-6.076). The expression of HO1 predicted shorter OS (*P* < 0.001, HR; 6.101, 95% CI; 2.832-13.143) and RFS (*P* < 0.001, HR; 3.476, 95% CI; 1.914-6.314). TNM stage was significantly associated with shorter OS (Log-rank, *P* = 0.010).

**Table 2:** Univariate Cox proportional hazards regression analysis for overall survival and relapse-free survival

**Characteristics**	**No.**	**OS**			**RFS**		
		**HR**	**95% CI**	***P***	**HR**	**95% CI**	***P***
Age, y, ≥ 50 (*vs*. < 50)	41/145	2.928	1.620-5.293	< 0.001	1.914	1.112-3.295	0.019
TNM stage, I	29/145	1		0.016	1		0.197
II	97/145	2.482	0.874-7.046	0.088	1.386	0.644-2.983	0.403
III and IV	19/145	5.154	1.614-16.457	0.006	2.288	0.902-5.799	0.081
Histologic grade, 1	49/145	1		0.014	1		0.087
2	67/145	1.218	0.575-2.579	0.607	1.147	0.598-2.198	0.680
3	29/145	2.818	1.293-6.139	0.009	2.077	1.027-4.203	0.042
HER2, positive (*vs*. negative)	40/145	2.765	1.525-5.012	< 0.001	2.596	1.511-4.461	< 0.001
ER, positive (*vs*. negative)	80/145	0.691	0.382-1.250	0.222	0.911	0.533-1.557	0.733
NGF, positive (*vs*. negative)	45/145	4.674	2.541-8.598	< 0.001	3.55	2.074-6.076	< 0.001
HO1, positive (*vs*. negative)	71/145	6.101	2.832-13.143	< 0.001	3.476	1.914-6.314	< 0.001
NGF/HO1, NGF^-^/HO1^-^	69/145	1		< 0.001	1		< 0.001
NGF^-^/HO1^+^	31/145	5.019	1.855-13.578	0.001	2.935	1.338-6.436	0.007
NGF^+^/anyHO1	45/145	9.717	4.003-23.586	< 0.001	5.41	2.754-10.625	< 0.001

We also performed additional survival analysis in the patients which received adjuvant chemotherapy or endocrine therapy. Among the patients who received systemic adjuvant chemotherapy, HER2 expression (Log-rank, OS; *P* < 0.001, RFS; *P* < 0.001), NGF expression (Log-rank, OS; *P* < 0.001, RFS; *P* < 0.001), and HO1 expression (Log-rank, OS; *P* < 0.001, RFS; *P* < 0.001) were significantly associated with shorter OS and RFS (Figure 2B). The age of the patients (*P* = 0.001) and TNM stage (*P* = 0.003) were significantly associated with shorter OS. Among the patients who received systemic adjuvant endocrine therapy, the age of the patients (Log-rank, OS; *P* < 0.001, RFS; *P* = 0.022), the expression of HER2 (Log-rank, OS; *P* = 0.007, RFS; *P* = 0.005), NGF (Log-rank, OS; *P* < 0.001, RFS; *P* < 0.001), and HO1 (Log-rank, OS; *P* < 0.001, RFS; *P* < 0.001) were significantly associated with both OS and RFS (Figure 2C). TNM stage was significantly associated with shorter OS (Log-rank, *P* = 0.024).

**Figure 3:**
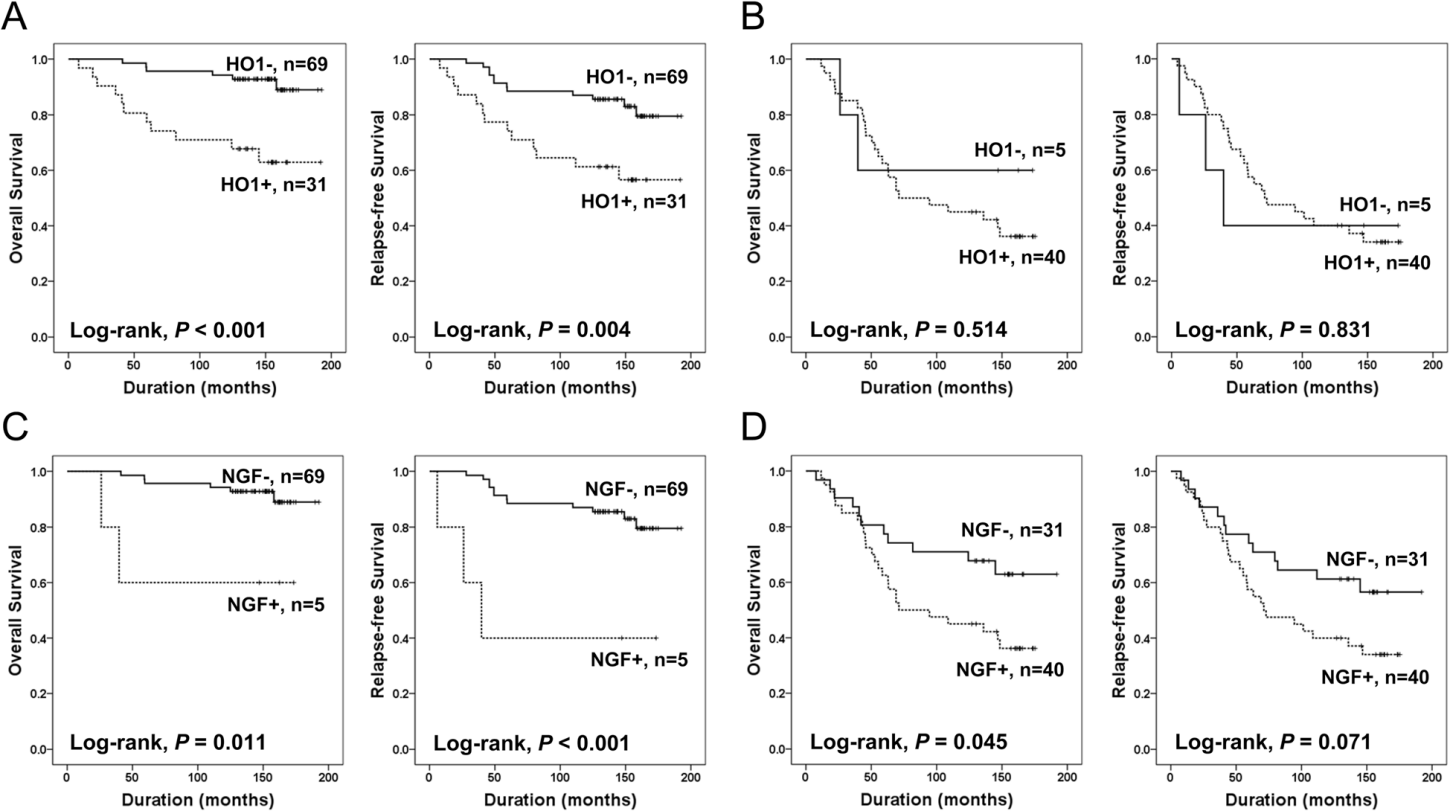
**Kaplan-Meier survival analysis between the expression of NGF and HO1.** Overall survival (OS) and relapse-free survival (RFS) according to the expression HO1 in the NGF-negative group **(A)** and the NGF-positive group **(B)**. OS and RFS according to the expression NGF in the HO1-negative group **(C)** and the HO1-positive group **(D)**.

**Figure 4:**
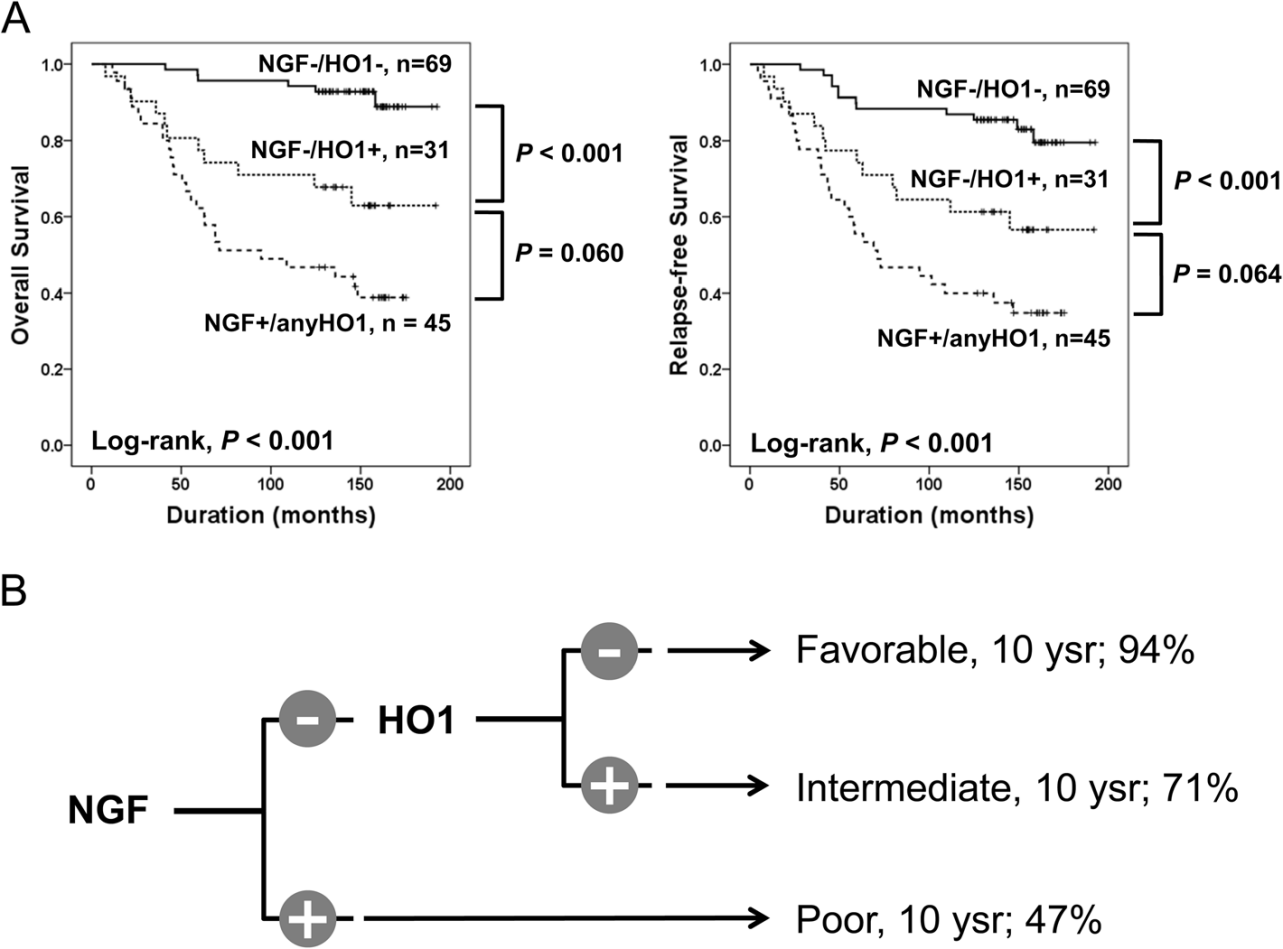
**Prognostic significance of the combined expression pattern of NGF and HO1. A.** Kaplan-Meier survival analysis for overall survival and relapse-free survival between the NGF^-^/HO1^-^, the NGF^-^/HO1^+^, and the NGF^+^/anyHO1 subgroups of breast carcinoma patients. **B.** An algorithm for the sub-grouping of breast carcinoma patients into three sub-groups according to the expression patterns of NGF and HO1. 10 ysr; ten-year survival rate.

Thereafter, to investigate the prognostic effect of the combined expression pattern of NGF and HO1 (NGF/HO1 expression), we analyzed the prognostic effect of the expression of one marker in two separate groups according to the positivity of another marker. In the NGF^-^ group, the expression of HO1 significantly associated OS (Log-rank, *P* < 0.001) and RFS (Log-rank, *P* = 0.004) (Figure 3A). However, HO1 expression did not affect for the survival of patients in NGF^+^ group (Log-rank, OS; *P* = 0.514, RFS; *P* = 0.831) (Figure 3B). However, NGF expression significantly associated with shorter OS of BRCA patients in both HO^-^ group (Log-rank, OS; *P* = 0.011, RFS; *P* < 0.001) and HO1^+^ (Log-rank, OS; *P* = 0.045, RFS; *P* = 0.071) group (Figure 3C and 3D). Based on these results, we divided the BRCA patients into three groups according to the NGF/HO1 expression pattern as shown in Figure 4. The NGF/HO1 expression was significantly associated with shorter OS (Log-rank, *P* < 0.001) and RFS (Log-rank, *P* < 0.001) (Figure 4A). The NGF^-^/HO1^-^ group showed favorable prognosis and the NGF^+^/anyHO1 group showed the poorest prognosis. The ten-year survival rate of the NGF^-^/HO1^-^ group, the NGF^-^/HO1^+^ group, and the NGF^+^/anyHO1 groups were 94%, 71%, and 47%, respectively (Figure 4B).

### NGF expression, HO1 expression, and NGF/HO1 expression is the independent, unfavorable prognostic predictor for overall survival in BRCA

The variables significantly associated with OS and RFS by univariate analysis were considered in the multivariate analysis. The variables considered in the multivariate analysis were age, TNM stage, histologic grade, and the expression of HER2, NGF, and HO1. Among the 145 BRCA patients, NGF expression was independent predictors of shorter OS and RFS. The patients with tumors expressing NGF had a 2.174-fold (95% CI; 1.073-4.404, *P* = 0.031) greater risk of shorter OS and a 3.042-fold (95% CI; 1.746-5.299, *P* < 0.001) greater risk of shorter RFS. The expression of HO1 (*P* < 0.001, HR; 4.847, 95% CI; 1.990-11.807) and TNM stage (overall *P* = 0.002) were also independent prognostic indicators of OS. The expression of HER2 was an independent prognostic predictor of RFS (*P* = 0.017, HR; 1.980, 95% CI; 1.132-3.464).To test the impact of the NGF/HO1 expression pattern on OS and RFS of BRCA patients, multivariate analysis was performed with the inclusion of NGF/HO1 expression instead of the individual expression of NGF and HO1. NGF/HO1 expression was also significantly associated with OS (overall *P* < 0.001) and RFS (overall *P* < 0.001) (Table 3).

**Table 3:** Multivariate Cox proportional hazards regression analysis for overall survival and relapse-free survival

**Characteristics**	**OS**			**RFS**		
	**HR**	**95% CI**	***P***	**HR**	**95% CI**	***P***
TNM stage,^*^ I	1		0.002			
II	3.542	1.241-10.109	0.018			
III and IV	7.933	2.441-25.787	< 0.001			
HER2,^*^ positive (*vs*. negative)				1.98	1.132-3.464	0.017
NGF,^*^ positive (*vs*. negative)	2.174	1.073-4.404	0.031	3.042	1.746-5.299	< 0.001
HO1,^*^ positive (*vs*. negative)	4.847	1.990-11.807	< 0.001			
NGF/HO1,^**^ NGF^-^/HO1^-^	1		< 0.001	1		< 0.001
NGF^-^/HO1^+^	6.542	2.381-17.979	< 0.001	3.5	1.574-7.778	0.002
NGF^+^/anyHO1	11.206	4.595-27.330	< 0.001	6.011	3.039-11.888	< 0.001

* The variables included in the multivariate analysis were age, TNM stage, and the expression of HER2, NGF, and HO1. ** The variables included in the multivariate analysis were age, TNM stage, HER2 expression, and the combined expression pattern of NGF and HO1 (NGF/HO1).

Among patients who received chemotherapy, TNM stage (overall *P* < 0.001), HO1 expression (*P* < 0.001), and NGF/HO1 expression (overall *P* < 0.001) were the independent prognostic predictor of OS. The expression of HER2 (*P* = 0.024) and NGF (*P* < 0.001), and NGF/HO1 expression (overall *P* < 0.001) were the independent prognostic indicators of RFS for BRCA patients. Among patients who received endocrine therapy, the age of the patients (*P* = 0.007), TNM stage (*P* = 0.022), HO1 expression (*P* < 0.001), and NGF/HO1 expression (overall *P* < 0.001) were independent prognostic indicators of OS for BRCA patients. The expression of HER2 (*P* = 0.029) and NGF (*P* < 0.001), and NGF/HO1 expression (overall *P* < 0.001) were independent prognostic indicators of RFS for BRCA patients.

## Discussion

In this study, we have investigated the immunohistochemical expression of NGF and HO1 in BRCA patients and demonstrated that the expression of NGF and HO1 were significantly associated with each other, and both have a significant association with HER2 expression, histologic grade, and latent distant metastasis. Moreover, the expression of NGF and HO1 was associated with shorter OS and RFS of BRCA by univariate analysis and multivariate analysis revealed the expression of NGF and HO1 as an independent prognostic indicator of OS for BRCA patients. Interestingly, the NGF/HO1 expression pattern was also a significant prognostic indicator of OS and RFS of BRCA patients by univariate and multivariate analysis. Especially, the patients with tumors expressing NGF had the shortest survival and the NGF^-^/HO1^-^ phenotype associated with favorable prognosis. This result suggests that both NGF and HO1 could be prognostic indicators and potential therapeutic targets for BRCA patients.

NGF is a neurotrophin, which controls development and survival of neuronal cells. In addition to its neurotrophic effect, NGF has been reported to be up-regulated in several malignant tumors [1,28]. Interestingly, NGF is not detected in normal breast epithelial cells and is not mitogenic for normal breast epithelial cells. In contrast, NGF is expressed in BRCA cells and stimulates growth of BRCA cells via an autocrine loop [7,29]. NGF stimulates proliferation and inhibits apoptosis of BRCA cells [2]. The pro-proliferative role of NGF in BRCA is mediated by activation of TrkA, p75^NTR^, and NFkB pathways [2,8]. Furthermore, NGF is involved in BRCA angiogenesis [3]. NGF increases the levels of secreted vascular endothelial growth factor in both human umbilical vein endothelial cells and BRCA cell lines [3]. Moreover, the precursor of NGF was overproduced in BRCA compared with benign breast tissue and involved in the stimulation of the invasion of BRCA cells [30]. TrkA overexpression promotes growth, migration, invasion, and survival of a BRCA cell line, enhances angiogenesis, and promotes metastasis of BRCA cells in mice [5]. Blocking of NGF with anti-NGF antibodies or small interfering RNA against NGF inhibited tumor growth and metastasis [6] and inhibitors for TrkA or p75^NTR^, downstream signaling targets of NGF, also had pro-apoptotic and anti-proliferative effects on BRCA cells [8]. The inhibition of the precursor of NGF by small interfering RNA inhibited invasion activity of BRCA cells [30]. Therefore, NGF has been suggested as a potential therapeutic target for the treatment of malignant tumors, especially in BRCA [6,8]. In our study, high NGF expression was associated with high histologic grade and the presence of distant metastasis, and predicted poorer survival of BRCA patients. The poor prognosis of the patients with NGF-expressing tumor might be related with the ability of NGF-TrkA signaling to induce chemoresistance [31]. Therefore, there is a possibility that NGF-targeted therapy with a combination of conventional chemotherapy could be helpful for BRCA patients. In agreement with our findings, the expression of the NGFRs TrkA and p75^NTR^ was associated with poor prognosis of pancreatic cancer patients [4]. In addition, similar results found in a recent report which demonstrated that the expression of NGFR significantly associated with the higher histologic grade of BRCA, suggesting the expression of NGFR as a potential indicator of poor prognosis [32]. In this large cohort study in BRCA, the expression of NGFR was negatively correlated with the expression of ER and indicative for the basal-like BRCA or luminal B subtypes [32]. Our result has shown a negative correlation between NGF expression and ER expression.

Despite recent advances in diagnosis and treatment modalities, BRCA remains the second leading cause of cancer-related death among women [33]. Recently developed targeted therapies, especially for the HER2, have led to the improvement of overall outcomes of BRCA patients [34-36]. Therefore, investigation of HER2 expression is crucial for the treatment of BRCA patients. However, the number of BRCA patients who benefit from targeted therapy is limited. The amplification of the *HER2* gene and overexpression of the HER2 protein has been detected in about 25% of BRCA and most patients with advanced BRCA with *HER2* gene amplification developed resistance to treatment [34,35]. However, the resistance mechanism to anti-HER2 treatments remains largely unexplained, and a new therapeutic approach for BRCA is needed. In our study, NGF expression was significantly correlated with HER2-positive status and the positivity of both NGF and HER2 predicted poor survival of BRCA patients. Similarly, a cooperative role of HER2 and NGF in the progression of BRCA has been previously reported [37], and NGF was suggested as a potential therapeutic target of BRCA [6]. However, when we separately analyzed the prognostic impact of NGF expression according to the expression status of HER2, NGF expression predicted poor survival of BRCA patients regardless of the positivity of HER2. NGF expression predicted poor OS in both the HER2-negative (Log-rank, *P* = 0.011) and HER2-positive subpopulations (Log-rank, *P* = 0.005). These results suggest the possibility that NGF has mechanism involved in the progression of BRCA which are independent of HER2-related mechanisms. Therefore, NGF-targeted therapy may be beneficial for BRCA patients in addition to the HER2-based conventional therapy.

In the NGF-TrkA signaling pathway, NGF is also known as an inducer of HO1 [22,23]. NGF increases HO1 expression through the receptor tyrosine phosphorylation pathway, and then HO1 causes the anti-apoptotic effect of NGF [22]. In neurodegenerative disease models, NGF protects cells against oxidative stress by inducing HO1 expression in a phosphatidylinositol 3-kinase-dependent manner [23]. These results suggest that there is a cooperative role between NGF and HO1 in cellular adaptation to stress and induction of resistance to death. When considering the tumor-progressing role of NGF, there is a possibility that HO1 is also involved in the progression of cancers. Our study has also showed a significant correlation between the expression of HO1 and NGF. 89% (40/45) of NGF-expressing BRCA co-expressed HO1. These results suggest the possibility that NGF and HO1 mediated pathways are involved in the progression of BRCA. However, interestingly, HO1 expression associated with shorter OS and RFS in the NGF-negative group, but not in the NGF-positive group. This finding suggests the possibility that HO1 may have its own role in the progression of BRCA-independent of an NGF- related mechanism.

The expression of HO1 is increased in various cancer cells compared with normal cells [17,18,38,39], which is associated with unfavorable prognosis of cancer patients [18]. The tumor-progressive role of HO1 is related to its roles in the inhibition of apoptosis [22], promotion of tumor angiogenesis [40,41], and chemoresistance [14]. HO1 also augments cancer cell migration and invasion by inducing MMP-9, CD147, and EGFR [18]. In the present study, the expression of HO1 was associated with unfavorable factors, distant metastatic relapse, higher histologic grade, and positive HER2 expression, and predicted shorter OS for BRCA patients. However, in contrast to our findings, there are conflicting reports that HO1 inhibits the proliferation and invasiveness of cancer cells [42-44], and that the expression of HO1 predicted favorable OS of colon cancer patients [20]. These conflicting findings may be related to the diverse roles of HO1 in various conditions or the status of the cells during tumorigenesis. HO1 may be protective for healthy cells in tumor-inducing injury; however, it could be tumor progressive in already developed tumors [45]. Therefore, further study is needed to explore the exact role(s) of HO1 and its possible correlation with NGF in BRCA tumorigenesis.

Another interesting finding of this study is that the combined expression pattern of NGF and HO1 is helpful for the prediction of the prognosis of BRCA patients. The patients with tumors expressing NGF had the shortest OS and RFS; furthermore, the patients with tumor which did not express NGF or HO1 showed the longest survival time. Multivariate analysis revealed NGF/HO1 expression as an independent prognostic indicator of OS and RFS. Therefore, this result suggests that the combined expression pattern of NGF and HO1 might be usable as a prognostic indicator for BRCA patients.

## Conclusions

In summary, the results of this study have shown that the expression of NGF and HO1 were significantly associated with each other and that the expression of both of them significantly correlated with unfavorable clinicopathological factors and predicted shorter survival of BRCA patients. Therefore, these results suggest the possibility that the NGF-HO1 pathway may be involved in breast carcinogenesis and progression. In addition, an algorithm for the sub-grouping of breast carcinoma patients into three sub-groups according to the expression patterns of NGF and HO1 also predicted survival of BRCA patients. Therefore, this result suggests the possibility that individual expression of NGF or HO1, and the combined expression pattern of NGF and HO1 could be the new prognostic indicator of BRCA patients.

## Abbreviations

BRCA: Breast carcinoma; CI: Confidence interval; ER: Estrogen receptor; HER2: Human epithelial growth factor receptor 2; HO1: Heme oxygenase-1; HR: Hazard ratio; LN: Lymph node; NGF: Nerve growth factor; NGFR: Nerve growth factor receptor; OS: Overall survival; PR: Progesterone receptor; RFS: Relapse-free survival; TMA: Tissue microarray; TrkA: Tropomyosin-related kinase A.

## Competing interests

The authors declare that they have no competing interests.

## Authors’ contributions

SJN, JSB, SHJ, BHP, HL, MJC, WSM, MJK and KYJ participated in the study design. JSB and UJ did the immunohistochemical staining. SJN, UJ, HSP, KSK, SHJ, HJY, MJC, MJK and KYJ were involved in data collection and data interpretation. SJN, KSK and KYJ participated in the statistical analyses. SJN, HSP, KSK, HJY, HL, BHP, WSM and KYJ wrote the manuscript. All authors read and approved the final manuscript.

## Pre-publication history

The pre-publication history for this paper can be accessed here:

http://www.biomedcentral.com/1471-2407/13/516/prepub
